# Behavioral adjustments to prior predation experience and food deprivation of a common cyprinid fish species vary between singletons and a group

**DOI:** 10.7717/peerj.7236

**Published:** 2019-07-08

**Authors:** Ya Wang, Shi-Jian Fu, Cheng Fu

**Affiliations:** Laboratory of Evolutionary Physiology and Behavior, Chongqing Key Laboratory of Animal Biology, Chongqing Normal University, Chongqing, China

**Keywords:** Starvation, Predator exposure, Inspection behavior, Behavioral adjustment, Predation risk

## Abstract

Fish often undergo predation stress and food shortages in nature, and living in groups may provide the ecological benefits of decreased predator risk but the costs of increased food competition. The main aim of the present study was to test whether the behavioral response of qingbo (*Spinibarbus sinensis*) to predators and/or starvation differed between a singleton and a group. We measured the locomotor activity and distance to a predator and/or food item of prior predator-experienced, starved, double-treated and control qingbo; the qingbo were tested both as singletons and in a group (five individuals). Fish from all groups showed increased activity when tested collectively compared to individually. The predator-experienced fish showed decreased locomotor activity to predators as an antipredator strategy when tested as singletons; however, increased locomotor activity occurred when tested in a group, which might be partially due to the decreased predator risk when living in a group and thus higher levels of boldness. As expected, starvation elicited increased activity indicating increased foraging willingness when tested in a group; however, the difference between starved and normal-fed fish was no longer significant when they were tested as singletons, possibly due to the increased predation risk and decreased food competition when living individually and higher behavioral variation among individual fish than among those in a shoal. Compared with the control fish, the double-treated fish showed no difference in activity when tested both individually and collectively (except a slower speed when tested in a group). The reason for the results from the singletons might be an offset of the effect of predator exposure and starvation. The reason for this difference in the group might be due to the impaired body condition indicated by a slower swimming speed as a consequence of severe stress. The present study demonstrated that behavioral adjustment was closely related to the size of the group, which might be due to differences in the predation risk and food competition.

## Introduction

Predators are one of the most important selective agents for behavioral traits in fish species (Bell, Henderson & Huntingford, 2010); therefore, altered behaviors, such as predator inspection and foraging under risk of predation, are frequent events during life histories in the wild (Fraser & Gilliam, 1992; Trimpop, 1994). Fish populations from high-predation habitats are sometimes bolder and show more spontaneous behavior than their conspecifics from low-predation habitats (Czeschlik et al., 2007; Bell, Henderson & Huntingford, 2010; Archard & Braithwaite, 2011). However, other studies have found that fish from high-predation populations show decreased activity and/or timidness and maintain a greater distance from predators (Magurran, 1986; Fu et al., 2015). Therefore, the behavioral adjustment of fish to predation pressure might be species specific. Studies have found that short-term predation stress treatment has a similar effect to the presence of predators in natural habitats; i.e., compared with nontreated conspecifics, predator-experienced fish showed alterations in behavior, metabolism or even escape ability under predation (Fu et al., 2017; Fu, Cao & Fu, 2019). Thus, investigations of behavioral adjustments among individuals with different prior predation experiences warrant study.

In addition to predation, starvation is another common stress event for fish species due to temporal and spatial fluctuations in food abundance (Kasumyan & Sidorov, 2010; Pang et al., 2016; Khan et al., 2018). For most fish species, the initial response to food deprivation is increased activity, which is indicative of greater searching behavior (Godin & Crossman, 1994; Miyazaki et al., 2000; Poulsen et al., 2010) and inevitably increases their predation risk. This conflict might shift to an energy-conservation behavioral adjustment due to a compromise between the predation risk and energy demand (Sogard & Olla, 1996), especially in a high-predation stress situation associated with low food availability (Killen, Marras & McKenzie, 2014). For example, adjustments in predator inspection and foraging behaviors in food-deprived fish in response to an increased predation risk may differ from the behaviors of their normal-fed conspecifics (Godlin & Crossman, 1994). In a previous study, we investigated the inspection behavior of qingbo (*Spinibarbus sinensis*), which is a common cyprinid fish species, and found that the starved fish displayed increased inspection behavior when measured without the predator; however, when measured in the presence of the predator, the starved fish showed an increased inspection frequency but shorter inspection duration, possibly due to the compromise between their energy needs and the predation risk (Tang et al., 2018). However, unlike the situation in the wild, no food item was provided in that study, which might have affected the behavioral adjustment of the starved fish. Thus, in the present study, the food item was considered with the aim of testing whether the behavior was altered by prior predator experience or food deprivation and whether both the prior predation experience and starvation had an interactive effect on behavior.

Living in groups has been suggested to result in the ecological benefits of decreased predation risk due to collective vigilance for predators, which is often referred to as the ‘many-eyes effect’ (Elgar, 1989; Godin, Classon & Abrams, 1988; Taraborelli et al., 2014). The so-called ‘confusion effect’ (living in larger shoals makes it more difficult for a predator to single out an individual prey, Landeau & Terborgh, 1986) and ‘dilution effect’ (the risk of an individual fish being caught diminishes as the number of individuals in the group increases, Foster & Treherne, 1981; Milinski et al., 1997) might also contribute to the decreased predation risk of living in a group. Furthermore, information communication by social interaction and cooperation among group members might also have a profound effect on foraging and inspection behaviors (Brown & Laland, 2002; Hesse et al., 2015a; Hesse et al., 2015b; Ioannou, Ramnarine & Torney, 2017; Schaerf, Dillingham & Ward, 2017). Thus, the behavioral adjustment of fish in a group might be different from those living as singletons. For example, competition for food may intensify with increased shoal size, and behavioral adjustments to food deprivation might also show differences between fish living as singletons and those in a group (Milinski et al., 1997; Ford & Swearer, 2013). To date, many studies have been conducted on fish groups (Morgan, 1988; Godin & Crossman, 1994; Hoare et al., 2004; Schaerf, Dillingham & Ward, 2017), although most studies have examined behavioral adjustments to predation and food shortages in individually measured fish (a few using dyads), possibly because extracting and analyzing behavioral traits is relatively easier in this context. In contrast, no comparison of possible differences in behavioral responses to food deprivation and (or) prior predator experience measured between a singleton and a group of fish is available for fish species, although more than half of fish species prefer to live in a group during at least some period of their life history (Shaw, 1978). Thus, the second and main aim of the present study was to test the hypothesis that behavioral adjustments to predation experience and food deprivation differ between singletons and a group.

To fulfill our goals, the qingbo (*Spinibarbus sinensis*), which is a freshwater cyprinid fish species that prefers group living and is mainly distributed in the upper Yangtze River and its tributaries, was selected as the experimental model. This fish forages frequently on low-nutrition vegetables, and the food resources in its natural habitat show profound fluctuations as a result of great environmental heterogeneity (Zeng et al., 2017). The predation risk is high in its natural habitat, and its potential predators include the southern catfish (*Silurus meridionalis*), which is a widespread piscivorous fish that was used as a predator in the study. Thus, the qingbo and southern catfish were selected with the aims of testing (1) whether the behavior of the qingbo was altered by prior predator experience or food deprivation and whether both prior predation experience and starvation had an interactive effect on behavior and (2) whether the behavioral adjustments of the qingbo to predation experience and food deprivation differed between singletons and groups.

## Materials & Methods

### Experimental fish and acclimation

Juvenile qingbo (Table 1) were collected from a local fish farm (Hechuan district, Chongqing, China) to guarantee that they were naive to fish predators and had not experienced starvation. Southern catfish (202.58 ± 1.71 g, 25.23 ± 0.86 cm; *N* = 20, repeatedly used) were obtained from a local aquatic product market (Shapingba district, Chongqing, China). Both fish species were kept in dechlorinated, fully aerated water tanks (1000 L, 200 × 100 × 50 cm) at a temperature of 20.0 ± 1.0 °C for one month before the experiments. The qingbo were fed frozen juvenile Chironomidae to satiation at 09:00 h. The southern catfish were fed to satiation with cutlets of freshly killed silver carp (*Hypophthalmichthys molitrix*) every two days at 09:00 h. The uneaten food and feces were removed by a siphon 1 h after feeding. All tanks had approximately 10% of the total water volume replaced daily. The photoperiod was approximately 12 L:12 D.

**Table 1:** Body mass, body length and sample size of the different groups of qingbo in the present study (mean ± SE).

			Fish number	
			Singleton	Group
*Experiment 1 (Predator treatment)*				
	Control group	*N*	20	20
		Body mass (g)	3.59 ± 0.28	3.59 ± 0.13
		Body length (cm)	5.7 ± 0.13	5.34 ± 0.07
	Predator-experienced group	*N*	20	20
		Body mass (g)	2.99 ± 0.14	3.63 ± 0.16
		Body length (cm)	5.11 ± 0.08	5.5 ± 0.09
*Experiment 2 (Starvation treatment)*				
	Control group	*N*	20	20
		Body mass (g)	3.13 ± 0.16	3.61 ± 0.14
		Body length (cm)	5.16 ± 0.1	5.45 ± 0.08
	Starved group	*N*	20	20
		Body mass (g)	3.42 ± 0.26	3.84 ± 0.27
		Body length (cm)	5.54 ± 0.14	5.6 ± 0.17
*Experiment 3 (Double treatment)*				
	Control group	*N*	20 (19)^a^	20
		Body mass (g)	3.36 ± 0.17	3.4 ± 0.18
		Body length (cm)	5.44 ± 0.09	5.39 ± 0.1
	Predator-experienced group	*N*	20	20
		Body mass (g)	3.42 ± 0.11	3.82 ± 0.18
		Body length (cm)	5.33 ± 0.05	5.57 ± 0.1
	Starved group	*N*	20 (19)	20
		Body mass (g)	3.22 ± 0.22	3.23 ± 0.23
		Body length (cm)	5.29 ± 0.21	5.56 ± 0.16
	Double-treated group	*N*	20 (16)	20
		Body mass (g)	2.75 ± 0.17	2.78 ± 0.13
		Body length (cm)	5.07 ± 0.13	5.12 ± 0.07

**Notes.**

a The number in parentheses indicates the actual number of videos that can be analyzed.

This study was approved by the Animal Care and Use Committee of the Key Laboratory of Animal Biology of Chongqing (Permit Number: Zhao- 20170912-02) and was performed in strict accordance with the recommendations in the Guide for the Care and Use of Animals at the Key Laboratory of Animal Biology of Chongqing, China.

**Figure 1:**
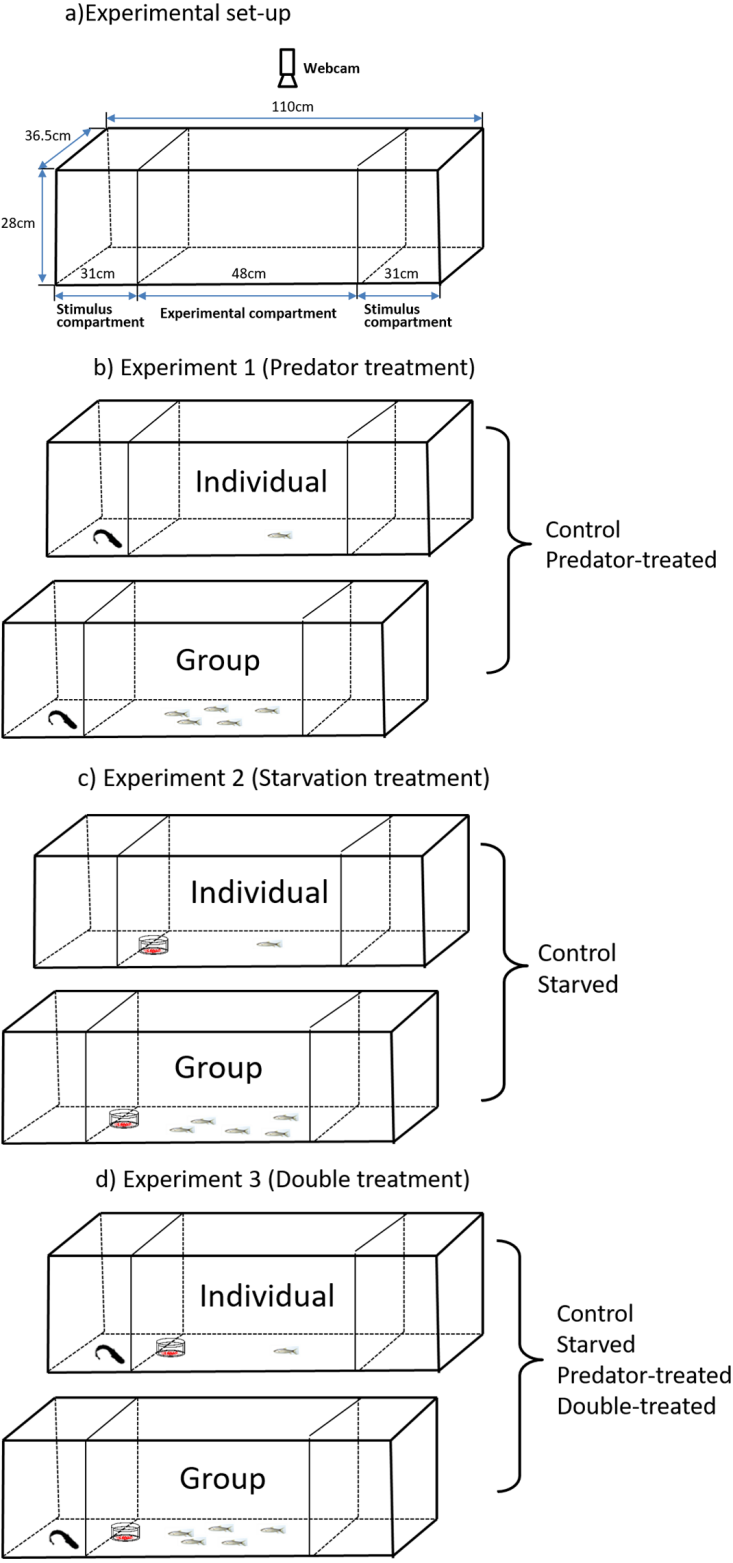
Experimental setup (A) for observation of spontaneous behaviors of the qingbo and the schematicdiagram (B, C, D) of the experimental design used in the present study.

### Experimental area

An aquarium (112 L, 110 × 36.5 × 28 cm, Fig. 1A) with all sides covered with white plastic sheets on the inner sides was used as the experimental area. The aquarium was divided into three compartments (one experimental compartment and two stimulus compartments) by a removable transparent partition. The experimental compartment (48 × 36.5 × 28 cm) was in the middle of the aquarium, and the two stimulus compartments (31 × 36.5 × 28 cm) were at both ends of the aquarium. These two stimulus compartments either housed a predator or were kept empty alternatively during the measurements. To permit visual and chemical communications between prey and predator, we cut two rows of holes with diameters of approximately 0.5 cm each in the bottom half of the removable transparent partitions. The behavior of the test fish (in the experimental compartment) was recorded by a webcam (above the aquarium; Logitech Webcam, Pro 9000) connected to a remote monitor, and the experimental aquarium was illuminated by carefully placed fluorescent tubes.

### Experimental protocol

#### Preprocess

The experimental qingbo (Table 1) were randomly divided into four groups for two weeks of preprocessing (i.e., a control, prior predator-experience, starvation and double-treated (starved fish with prior predation treatment) groups). The fish in the control group were maintained the same as those in the preacclimation period for two weeks. The fish in the predator-experience group were maintained similarly to the fish in the control group except that the former were housed together with a caged southern catfish (the effect of a caged predator on routine activities is similar to that of direct predation according to Liu et al. (2016); thus, we used this treatment to avoid ethical issues with experimental animal use). The fish in the starved group were deprived of food for two weeks. The fish in the double-treated group experienced the same predator exposure treatment but were also deprived of food during the two-week treatment period. All qingbo except the starved individuals fasted for two days prior to any of the experimental measurements (Tang et al., 2018; Xiong et al., 2018).

#### Experiment 1 (Predator treatment)

One southern catfish was transferred to the stimulus compartment (alternatively) of the aquarium, whereas the compartment on the other side remained empty (see details in Fig. 1B). At the same time, either one individual or a group of five individuals from the predator-experienced group was gently captured with a net in their holding tank, transferred into a plastic beaker without exposure to air and released carefully into the experimental compartment. The entire process was completed within 1 min. Then, the fish were acclimated for 10 min (Lacasse & Aubin-Horth, 2012; Killen et al., 2016), and the behavior of the qingbo was monitored for 20 min by webcam at a rate of 15 frames per second. For the fish in the control group, the same process described for the fish in the predator-experienced group was performed except for the lack of predation experience. Twenty replicate experiments were performed for each group (predator-experienced in singleton, predator-experienced in a group, control in singleton and control in a group), and no qingbo was reused in this study (Table 1). After measurement of each test fish, both the body mass and length of each fish were measured. For the fish tested in a group, after monitoring their behavior for more than 20 min, one of the five fish was randomly captured with a net as the focus fish for measurement of the body mass and length, followed by the other four fish. In this way, the webcam would record which fish was the focus fish (see details in the Data collection and video analysis section). After each measurement, the experimental tanks were cleaned and refilled with fresh dechlorinated water. The water temperature conditions were controlled at 20 °C (±1), and all experiments were conducted between 8:00 and 18:00. (Tang et al., 2018; Xiong et al., 2018).

#### Experiment 2 (Starvation treatment)

The measurement procedure in experiment 2 (see details in Fig. 1C) was similar to that in experiment 1 except that the treatment fish were from the starved group. Furthermore, no predator was contained in the stimulus compartment; instead, frozen juvenile Chironomidae packaged in two transparent oppositely placed culture dishes (diameters 10 and 12 cm) were placed against the partition. The qingbo could see the juvenile Chironomidae though the transparent culture dishes but could not obtain them.

#### Experiment 3 (Double treatment)

The procedure was the same as those described above, but both the predator and food items were present during the measurements (see details in Fig. 1D). In addition to the fish from the double-treated group, the fish from the predator-experienced group (without starvation treatment) and starved group (without predation treatment) were also measured with both predator and food items present.

### Data collection and video analysis

The trajectory of each frame was digitized with the idTracker software (Pérez-Escudero et al., 2014; Tang et al., 2017), which automatically tracked the position of each fish in each trial and provided all x and y coordinates for each fish in each video (Miller & Gerlai, 2012; Killen et al., 2016). When the fish were measured in the group, only the data from the focus fish were recorded and analyzed. The focus fish was removed first from the experimental area; thus, the fish that was the first to lose its coordinates was the focus fish for analysis of the videos with idTracker. The raw trajectories were smoothed using a weighted average with a window width of 0.5 s, because the trajectories were noisy due to changes in body shape during recording and errors from the tracking device (Miller & Gerlai, 2012). Of the 320 videos, six failed to be digitized by idTracker and were excluded from the analyses (see details in Table 1).

### Parameter calculations

In this study, we measured locomotor activity indicated by the swimming speed and percent time moving (PTM) of both individuals and groups of five individuals with different treatments. Furthermore, the distance to the predator or food item was also measured.

The x and y coordinates were used for parameter calculations. First, instantaneous speed (*v*, cm s^−1^) was measured as follows:

(1) \begin{eqnarray*}v(t)=\sqrt{(x(t)-x(t-1))^{2}+(y(t)-y(t-1))^{2}}/d\end{eqnarray*}

where *x* (*t*), *x* (*t-1*) and *y* (*t*), *y* (*t-1*) are the *x* and *y* coordinates of the measured fish at time *t* and the previous frame (*t-1*) and *d* is the length of the time interval. The fish were considered moving when the swimming speed was greater than 1.75 cm s^−1^; otherwise, the fish were considered to be resting (Tang et al., 2017). Then, the speed while moving (cm s^−1^) was calculated as the average instantaneous speed of individuals when their instantaneous speeds were higher than 1.75 cm s^−1^. The PTM (%) was calculated as the percentage of time when an individual’s instantaneous speed was higher than 1.75 cm s^−1^.

The distance to the predator or food (cm) was calculated as the average distance between the qingbo and the transparent partition (the side with the predator and (or) food, respectively).

### Statistical analysis

The statistical analyses were performed with the Statistics Program for Social Sciences (SPSS) Statistics 17.0 (SPSS, Chicago, IL, USA). The effects of prior experience (predator exposure, starvation or double treatment) and fish numbers (individual vs group) on all three measured variables were determined using a two-way analysis of covariance (ANCOVA) with body mass as a covariate. The differences in any variables measured between the treatment and control groups as well as between an individual and group of fish were compared by post-hoc tests if necessary. The statistical significance level was corrected to *P* < 0.017 (i.e., 0.05/3 variables).

## Results

### Experiment 1 (Predator treatment)

**Figure 2:**
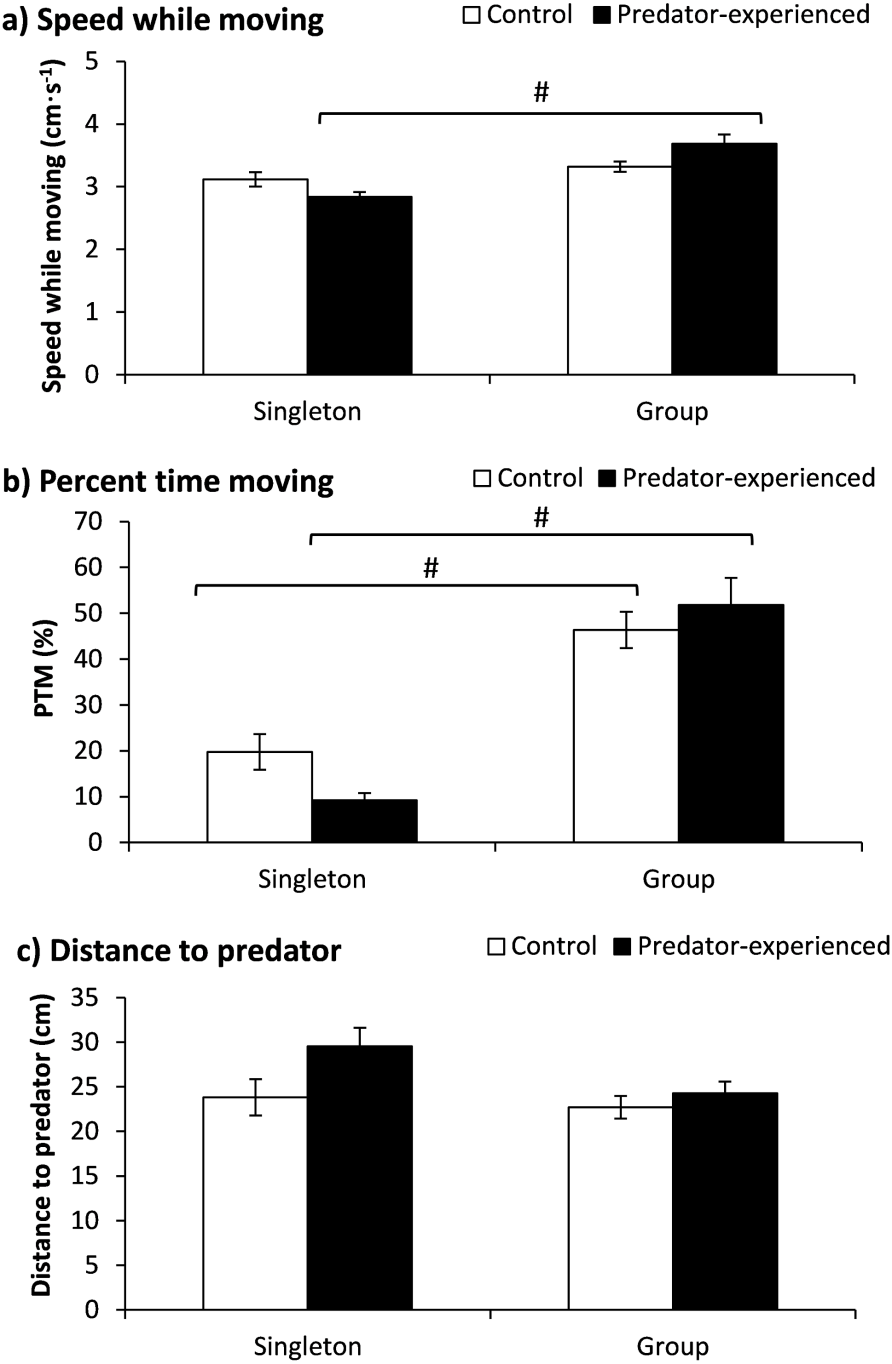
The effects of prior predator exposure treatment and the tested fish number on the speed while moving (A), percent time moving (B) and distance to the predator (C) in qingbo (mean ± S.E.). *N* = 20. # indicates a significant difference between singletons and groups of fish within either the control or predator-experienced group (*P* < 0.017).

The fish number showed a significant effect on speed while moving, but the treatment had no significant effect on speed; additionally, an interaction existed between the treatment and fish number (see detailed significance levels in Table 2). The predator-experienced fish tested in a group showed significantly higher speeds while moving than those tested as singletons (*P* < 0.001; Fig. 2A). The fish number exhibited a significant effect on the PTM (Table 2). Compared with the singleton fish, the fish in a group showed a significantly higher PTM regardless of whether they had or lacked predator experience (*P* < 0.001) (Fig. 2B). Neither the treatment nor the fish number showed any effect on the distance to the predator (Table 2).

**Table 2:** The effect of treatment and the test number on the measured variables in juvenile qingbo based on two-way multivariate analysis of variance (MANCOVA).

	Covariate effect	Treatment effect	Fish number effect	Interaction effect
*Experimental 1 (Predator treatment)*				
Speed while moving	*F*_1.75_ = 0.027	*F*_1.75_ = 0.139	*F*_1.75_ = 22.234	*F*_1.75_ = 8.297
	*P* = 0.869	*P* = 0.711	*P* < 0.001^*^	*P* = 0.005^*^
Percent time moving	*F*_1.75_ = 0.199	*F*_1.75_ = 0.285	*F*_1.75_ = 66.473	*F*_1.75_ = 3.280
	*P* = 0.657	*P* = 0.595	*P*<0.001^*^	*P* = 0.074
Distance to predator	*F*_1.75_ = 0.357	*F*_1.75_ = 4.779	*F*_1.75_ = 3.771	*F*_1.75_ = 1.712
	*P* = 0.552	*P* = 0.032	*P* = 0.056	*P* = 0.195
*Experiment 2 (Starvation treatment)*				
Speed while moving	*F*_1.75_ = 0.877	*F*_1.75_ = 3.778	*F*_1.75_ = 12.893	*F*_1.75_ = 1.505
	*P* = 0.352	*P* = 0.056	*P* = 0.001^*^	*P* = 0.224
Percent time moving	*F*_1.75_ = 0.434	*F*_1.75_ = 9.014	*F*_1.75_ = 93.797	*F*_1.75_ = 1.843
	*P* = 0.512	*P* = 0.004^*^	*P*<0.001^*^	*P* = 0.179
Distance to food	*F*_1.75_ = 0.176	*F*_1.75_ = 0.265	*F*_1.75_ = 3.648	*F*_1.75_ = 0.001
	*P* = 0.676	*P* = 0.608	*P* = 0.060	*P* = 0.979
*Experiment 3 (Double treatment)*				
Speed while moving	*F*_1.145_ = 0.005	*F*_3.145_ = 11.239	*F*_1.145_ = 5.569	*F*_3.145_ = 4.276
	*P* = 0.946	*P*<0.001^*^	*P* = 0.020	*P* = 0.006^*^
Percent time moving	*F*_1,145_ = 1.792	*F*_3,145_ = 7.390	*F*_1,145_ = 119.753	*F*_3,145_ = 2.834
	*P* = 0.183	*P*<0.001^*^	*P*<0.001^*^	*P* = 0.040
Distance to predator (or food item)	*F*_1,145_ = 2.278	*F*_3,145_ = 0.783	*F*_1,145_ = 1.722	*F*_3,145_ = 1.600
	*P* = 0.133	*P* = 0.505	*P* = 0.192	*P* = 0.192

**Notes.**

* Significant (*P* < 0.017, i.e., 0.05/3).

### Experiment 2 (Starvation treatment)

**Figure 3:**
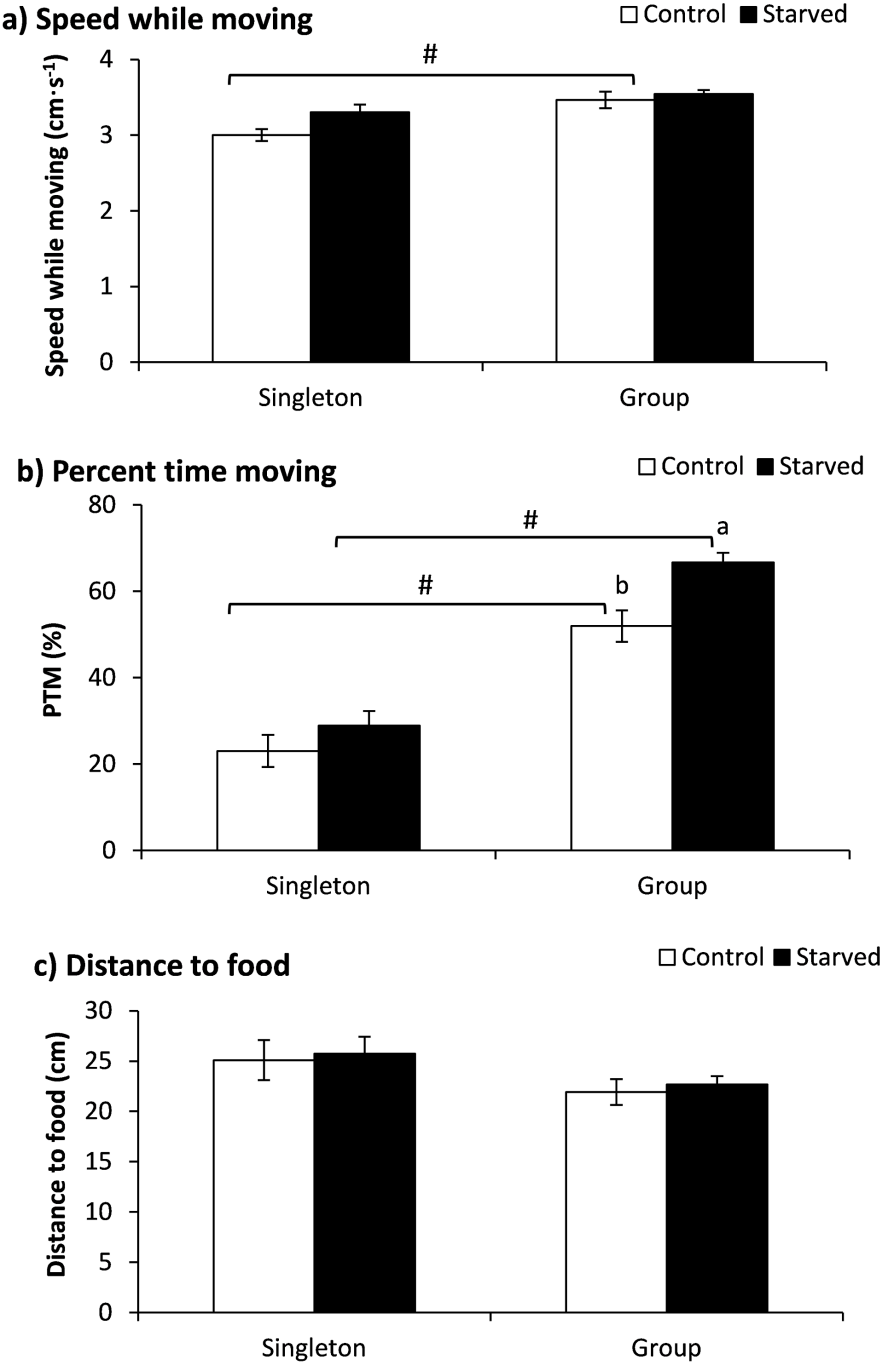
The effects of starvation and the tested fish number on the speed while moving (a), percent time moving (b) and distance to food (c) in qingbo (mean ± S.E.). *N* = 20. (A and B) different letters indicate a significant difference between the control and starved groups within each tested fish number (*P* < 0.017). # indicates a significant difference between singletons and groups of fish within either the control or starved group (*P* < 0.017).

The fish number showed a significant effect on speed while moving (Table 2), and the fish of the control group tested in a group showed higher speeds than those tested as singletons according to a further *t*-test (*P* = 0.002) (Fig. 3A). Both the treatment and fish number exhibited significant effects on the PTM (Table 2). The PTM was significantly higher when tested in the group than when tested in singletons independent of the food deprivation treatment (*P* < 0.001). Furthermore, the starved fish showed a higher PTM when tested in a group (*P* = 0.001) (Fig. 3B). Neither the treatment nor the fish number showed any effect on the distance to food items (Table 2).

### Experiment 3 (Double treatment)

**Figure 4:**
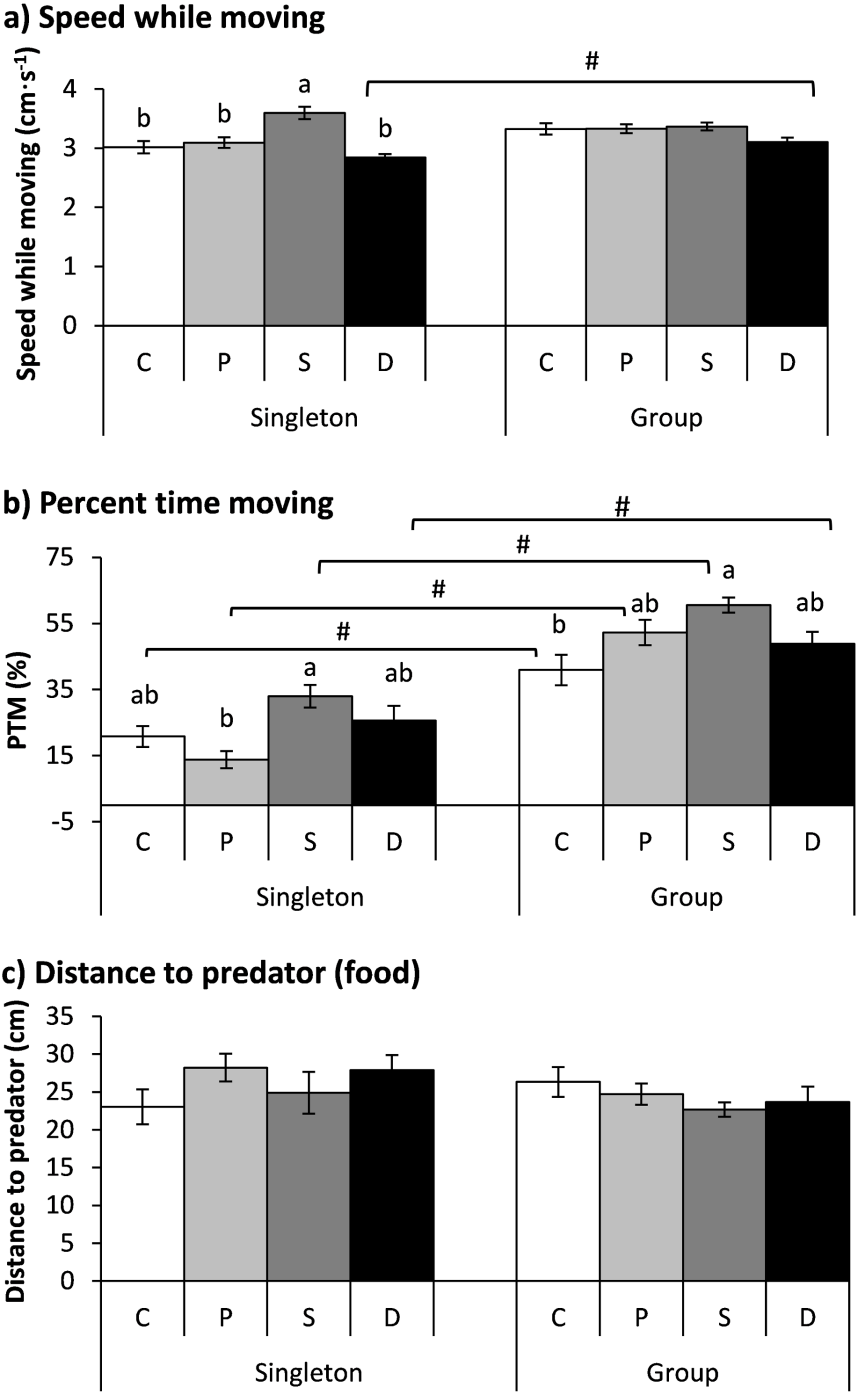
The effects of the double treatment and tested fish number on the speed while moving (A), percent time moving (B) and distance to the predator (food) (C) in qingbo (mean ± S.E.). *N* = 20, except *N* = 19 for the control and starved group and *N* = 16 for the double-treated group when tested as singletons. C: control group; P: predator-experienced group; S: starved group; D: double-treatment group.a and b: different letters indicate significant differences within different treatment groups (control, predator-experienced, starved and double-treatment groups) within each tested fish number (*P* < 0.017). # indicates significant differences between singletons and groups of fish within different treatment groups (control, predator-experienced, starved and double-treatment groups; *P* < 0.017).

The treatment showed a significant effect on speed while moving, but the fish number had no significant effect on speed; additionally, an interaction existed between the treatment and fish number (Table 2). The speed of the starved fish was significantly higher than that of the other three groups when the fish were tested as singletons (*P* < 0.001). Furthermore, the double-treated fish showed higher speeds when tested in a group than when tested as singletons (*P* = 0.011; Fig. 4A).

Both the treatment and fish number showed significant effects on the PTM (Table 2). All four groups showed higher PTMs when measured in a group than when measured as singletons (*P* < 0.017). Compared with the control fish, the double-treated fish showed no significant difference when measured as singletons or in a group. The PTM of the starved fish was significantly higher than that of the predator group when tested as singletons and was higher than that of the control group when tested in a group (*P* < 0.001; Fig. 4B).

Neither the treatment nor the fish number showed any effect on the distance to the predator (Table 2).

## Discussion

### Fish in a group showed higher activity

In this study, the increase in activity when tested in a group compared to that of the singletons was independent of the treatment. Furthermore, fish from all four groups showed a similar pattern of change between a single fish and a group of fish. The increase in activity measured in a group compared to that measured in singletons has been previously documented in fish species (Fu, 2016). The mechanisms might involve increased foraging behavior as a consequence of decreased predation risk and increased resource competition when at a shoal in a group rather than as singletons, as mentioned in the introduction (Milinski et al., 1997; Ford & Swearer, 2013; Hesse et al., 2015b), although the increase of activity itself may lead to a higher predation risk (Martel & Dill, 1995).

Notably, the speed and PTM values showed little change among all three experiments. This result suggests that the change in the structure of the area (i.e., placement of food items and predators) had little effect on activity within the three experiments. Furthermore, this result also meant that the measurements of the variables were consistent across the experiments.

### Single fish and groups of fish showed opposite behavioral adjustments to predators

When measured as singletons, prior predator experience resulted in a 57% lower PTM (*P* = 0.016). The swimming speed also showed a 9% decrease (*P* = 0.054). This result suggests that predator-experienced qingbo may downregulate their activity as their antipredator strategy when measured as singletons. The 24% greater distance to the predator (*P* = 0.055) also suggested that the predator-experienced qingbo were less bold. Similar results have been found in fish species such as rainbowfish (*Melanotaenia duboulayi*) (Brown & Warburton, 1999) and minnow (*Phoxinus phoxinus*) (Magurran, 1986). However, when tested in a group, both the swimming speed (*P* = 0.038) and the PTM of the predator-experienced qingbo had higher values than those of the naive qingbo. Furthermore, the difference in the distance to the predator disappeared. Although the differences between the control and predator-experienced groups did not reach significant levels when the significance level was adjusted to 0.017, an obvious opposite behavioral adjustment to the presence of predators between groups and single fish was shown in this study. Although the mechanisms require further investigation, possible explanations may include a decreased predator risk and hence greater boldness (Godin, Classon & Abrams, 1988; Taraborelli et al., 2014), increased competition for food and other resources (Ford & Swearer, 2013) and more inclination toward social interaction and mimicking of the behavior of neighbors under predation risk conditions (Ioannou, Ramnarine & Torney, 2017; Schaerf, Dillingham & Ward, 2017).

### Starved fish showed increased activity

As anticipated, starvation resulted in elevated activity, most likely due to the increase in foraging or predator inspection behaviors as a consequence of shortened energy storage that increased appetite, as demonstrated previously (Godin & Crossman, 1994; Miyazaki et al., 2000; Tang et al., 2018). However, the adjustment was much more profound when tested in a group (fish in a group showed a 28% increase in the PTM, whereas singleton fish showed an 18% increase in the PTM). As previously mentioned, one reason for this result might be that the higher predation risk restrained the foraging or exploration behavior of the starved fish measured as singletons. Furthermore, enhanced food competition in the group might have aggravated the behavior between starved and normal-fed qingbo (Ford & Swearer, 2013). The distance to the food item was not different between the starved and normal-fed qingbo, which was not consistent with our expectation. As the food was placed within two oppositely placed transparent culture dishes, the results of the present study suggested that qingbo may not have obtained the necessary information from the food items and hence exhibited no response to them.

### Double-treated fish showed no adjustment by either offsetting both treatments (singleton) or impairing the body condition (group)

Interestingly, although both predator experience and food deprivation had a profound effect on the activity of the qingbo, the double-treated qingbo showed no difference in activity when tested as singletons relative to that of the fish from the control group. This result is reasonable for fish measured as singletons because a predator-elicited decrease in activity might offset the starvation-elicited increase in activity. Furthermore, unexpectedly, neither of the two tested variables of the double-treated qingbo group changed compared with those of the control group. A possible explanation for this result might be that food deprivation and predator exposure imposed too much stress on the experimental fish. Thus, the physiological condition of the body might have been impaired as a consequence of sustained elevation of the stress response (Sapolsky, 2002). An impaired body condition was supported by the swimming speed, which was lower in the double-treated fish than in the control fish when measured in a group.

## Conclusions

In conclusion, fish in a group showed increased activity, mainly by spending more time moving, possibly due to a decreased predation risk and increased resource competition. Compared with naive fish, the predator-experienced fish in singletons and groups showed a distinctly opposite strategy for activity in response to their predators. When subjected to food deprivation, the starved fish exhibited increased activity due to carrying out more foraging behavior as a result of their upregulated appetite; the increased foraging behavior was restrained to some extent when tested individually. The double-treated fish showed no variation in their behavioral responses to the different treatments when tested as singletons, possibly due to the offset between the two treatments; however, the lack of change in behavior in a group of fish was unanticipated and possibly occurred due to overstress and a poor body condition. Nevertheless, the results of the present study suggested that the behavioral strategies used to address food deprivation and predator exposure varied between singletons and groups of fish and that the combination of food deprivation and predator experience was far more complex than originally thought.

##  Supplemental Information

10.7717/peerj.7236/supp-1 Data S1 raw data of the Experiment 1-Predator treatment, Experiment 2-Starvation treatment and Experiment 3-Double treatment in qingbo The raw data of body size and behavioral parameters of each fish was given in this file.

## Additional Information and Declarations

### Data Availability

The following information was supplied regarding data availability: The raw measurements are available as a Supplemental File.
